# Agro-Industrial Wastes as Potential Substrates for Rhamnolipid Production by *Pseudomonas aeruginosa* USM-AR2

**DOI:** 10.21315/tlsr2024.35.1.3

**Published:** 2024-03-30

**Authors:** Mohd Shafiq Nasir, Ahmad Ramli Mohd Yahya, Nur Asshifa Md Noh

**Affiliations:** School of Biological Sciences, Universiti Sains Malaysia, 11800 USM Pulau Pinang, Malaysia

**Keywords:** Biosurfactant, Rhamnolipid, Agro-Industrial Waste, *Pseudomonas aeruginosa*, Submerged Culture Fermentation, Biosurfaktan, Rhamnolipid, Sisa Agro-Industri, *Pseudomonas aeruginosa*, Fermentasi Kultur Tenggelam

## Abstract

Rhamnolipid has gained much attention in various fields owing to its distinctive functional properties compared to conventional chemical surfactants, which are mostly derived from petroleum feedstock. Production cost is one of the main challenges in rhamnolipid production, particularly when using refined substrates. One possible solution is to use agro-industrial wastes as substrates for rhamnolipid production. This is a promising strategy due to their abundance and commercially low value, while simultaneously alleviating an agro-industrial waste management problem in the environment. This study aims to evaluate agro-industrial wastes from local crops as possible low-cost alternative substrates for rhamnolipid production by a local isolate, *Pseudomonas aeruginosa* USM-AR2. Various liquid wastes, namely sugarcane molasses, rice washing water, overly mature coconut (OMC) water, empty fruit bunch (EFB) steam effluent, palm sludge oil (PSO) and palm oil mill effluent (POME) were screened as the main carbon source supplementing mineral salt medium (MSM) in the fermentation of *P. aeruginosa* USM-AR2. Batch fermentation was carried out in a shake flask system, agitated at 200 rpm and incubated at room temperature, 27 ± 2°C for 120 h. Among the substrates tested, PSO exhibited the highest biomass at 20.78 g/L and rhamnolipid production at 1.07 g/L. This study has shown the potential of agro-industrial wastes in Malaysia as an alternative resource for rhamnolipid production, transforming them into value added products, while reducing the amount of wastes discharged into the environment.

## Abstract (ms)

Rhamnolipid telah menjadi tumpuan pelbagai bidang disebabkan fungsi dan ciri-ciri tersendiri berbanding surfaktan kimia konvensional yang kebanyakannya diperoleh daripada sumber petroleum. Kos penghasilan merupakan salah satu daripada cabaran utama dalam penghasilan rhamnolipid, terutamanya apabila menggunakan substrat terproses. Salah satu penyelesaian adalah dengan menggunakan sisa agro-industri sebagai substrat untuk penghasilan rhamnolipid. Ini merupakan strategi berpotensi berdasarkan jumlahnya yang banyak dan nilai komersial yang rendah, di samping mengurangkan masalah pengurusan sisa agro-industri dalam alam sekitar. Kajian ini bertujuan untuk menilai potensi sisa agro-industri daripada tanaman tempatan sebagai substrat alternatif kos rendah untuk penghasilan rhamnolipid oleh pencilan tempatan, *Pseudomonas aeruginosa* USM-AR2. Pelbagai sisa cecair iaitu molases tebu, air basuhan beras, air kelapa tua, wap efluen tandan kosong (EFB), enap cemar minyak sawit (PSO), dan efluen kilang kelapa sawit. (POME) telah disaring sebagai sumber karbon utama yang melengkapi medium garam mineral untuk fermentasi *P. aeruginosa* USM-AR2. Fermentasi kelompok telah dijalankan di dalam sistem kelalang goncangan, diaduk pada 200 rpm dan dieram pada suhu 27 ± 2°C selama 120 jam. Antara substrat yang telah diuji, enap cemar minyak sawit menunjukkan biojisim tertinggi pada 20.78 g/L dan penghasilan rhamnolipid sebanyak 1.07 g/L. Kajian ini menunjukkan potensi sisa agro-industri di Malaysia sebagai sumber alternatif untuk penghasilan rhamnolipid, mengubah sisa agro-industri kepada produk dengan nilai tambah di samping mengurangkan jumlah sisa yang dilepaskan ke alam sekitar.

**Highlights**

- Malaysian agro-industrial waste substrates can potentially be used for rhamnolipid production in *Pseudomonas aeruginosa* USM-AR2 fermentation.
- Oil palm-based agro-industrial waste substrates gave higher rhamnolipid production compared to saccharide substrates, with palm sludge oil giving the highest production of rhamnolipid.
- Agro-industrial waste can serve as a cheap alternative substrate for rhamnolipid production while simultaneously minimising waste disposal problem for environmental management.

## INTRODUCTION

Biosurfactants are surface-active amphiphilic compounds produced by various microorganisms including bacteria, yeasts and fungi. The unique properties of biosurfactants include biodegradability, better environmental compatibility, strong emulsification towards hydrophobic compounds, low toxicity, stability, antimicrobial activities and synthesised from renewable sources. These properties make them valuable in various industrial applications such as food processing, environmental bioremediation, oil and gas processing, pharmaceutical production and agricultural applications. The most widely studied biosurfactant is rhamnolipid, an anionic glycolipid biosurfactant produced mainly by the Gram-negative bacterium, *Pseudomonas aeruginosa* as a secondary metabolite (Varjani *et al*. 2021). Rhamnolipid consists of one or two head groups of rhamnose moiety linked to a fatty acid tail, such as β-hydroxydecanoic acid, producing two forms, i.e., mono-rhamnolipid and di-rhamnolipid (Wu *et al*. 2019). Previous report has shown the ability of *P. aeruginosa* in utilising a variety of water soluble and hydrophobic carbon source as substrates for rhamnolipid production (Liu *et al*. 2019).

Due to its similarity in function to its chemically synthesised counterparts, the global market for biosurfactants was estimated at USD3.99 billion in 2016 and predicted to reach USD5.52 billion by 2022 (Singh *et al*. 2019; Wang *et al*. 2020). However, the increasing awareness on the risk associated with synthetic surfactants has caused the surfactant industry to slowly turn towards producing the more environmentally friendly biosurfactants. Biosurfactants are synthesised in fermentation processes using refined substrates that are costly, driving production cost up and consequentially making it economically unattractive. Manufacturers must overcome this hurdle to make biosurfactants commercially competitive (Tan & Li 2018).

An alternative approach needs to be taken towards efficient biosynthesis and downstream processing. The agricultural sector has been one of Malaysia’s main sources of economy. Malaysia has heavily invested in the development of agriculture, especially in plantation to improve crops production and efficiency in processing (Bujang & Bakar 2019). Nonetheless, there is ample room for improvement in the agro-industrial waste management, namely for liquid wastes or slurries. Using agro-industrial wastes as alternative substrates promises a cheaper way to produce biosurfactants due to their abundance and low commercial value. Many agro-industrial waste substrates contain vital nutrients to support microbial growth and biosurfactant production. Moreover, utilisation of agro-industrial wastes may also alleviate the problem of waste discarded into the environment.

This study evaluates wastes from common local crops that can be used in rhamnolipid production by *P. aeruginosa* USM-AR2. These are sugarcane molasses, rice washing water, overly matured coconut (OMC) water, empty fruit bunch (EFB) steam effluent, palm sludge oil (PSO) and palm oil mill effluent (POME). Sugarcane molasses is a sugar-containing waste by-product obtained during the final sugar refinement process. Rice washing water is a cloudy, starch-rich liquid waste obtained from washing rice before cooking (He *et al*. 2019). The OMC water liquid waste substrate is obtained during the extraction of coconut kernel to produce coconut milk. OMC water offers higher content in minerals, sucrose and protein compared to that of younger stages. There is no report to date on OMC water used as a fermentation substrate, other than mature coconut water in the production of nata de coco (Tan *et al*. 2019; Santosa *et al*. 2020). EFB steam effluent is collected in residual oil re-extraction from shredded empty fruit bunch using steam (Akhbari *et al*. 2020). Due to its fibrous lignocellulosic nature, EFB is usually favoured in solid fermentation by filamentous organisms that can degrade lignocellulose (Aditiawati *et al*. 2019; Mohd Luthfi *et al*. 2020). Many reports have discussed the potential of solid empty fruit bunch as a potential substrate in solid state fermentation as reported by Musa *et al*. (2017) and Sinjaroonsak *et al*. (2019). Nonetheless, no study has been published on its steam effluent. PSO is a floating residual oil, separated during the initial stage of POME discharge to the pond (Muanruksa *et al*. 2019; Muanruksa & Kaewkannetra 2020). POME is the final liquid discharge that is generated from oil extraction, washing and cleaning processes in the mill, containing cellulosic material, fat, oil and grease depending on the raw material quality (Hermansyah *et al*. 2018; Bukhari *et al*. 2020).

This is the first comparative study on the production of rhamnolipid using several agro-industrial wastes. The novelty of this study highlights the agro-industrial substrates which have not been reported elsewhere for rhamnolipid production, such as rice water, OMC, PSO and EFB steam effluent. Following the screening of the six agro-industrial substrates in shake flask fermentation, substrates that gave higher rhamnolipid production were tested at different substrate concentrations to observe if higher nutrient results in higher rhamnolipid production.

## MATERIALS AND METHODS

### Bacterial Culture

The bacterium used in this study was *P. aeruginosa* USM-AR2, originally isolated from a local crude oil sample. It was subsequently identified as a hydrocarbon-utilising and rhamnolipid-producing bacterium (Md Noh *et al*. 2014). The strain was maintained in 40% (v/v) glycerol at −20°C for long term storage.

### Waste Substrate Collection

Various agro-industrial wastes were collected from several locations. Sugarcane molasses was bought from Effective Microorganism Research Organisation (EMRO) Malaysia Sdn Bhd, Skudai, Johor, a company that supplies sugarcane molasses as fertiliser. OMC water was freshly collected from a local wet market in Gelugor, Pulau Pinang. Rice washing water was collected from nearby houses in Batu Uban, Pulau Pinang. EFB steam effluent, PSO and POME were collected form MALPOM Palm Oil Mill Industries Berhad in Jawi, Pulau Pinang. All substrates were stored at 4°C and brought to room temperature before use except for OMC water which was used fresh. These waste substrates were used as the main carbon source for submerged fermentation of *P. aeruginosa* USM-AR2 to produce rhamnolipid.

### Culture Medium

Mineral salt medium (MSM) for the cultivation of *P. aeruginosa* USM-AR2 in submerged fermentation was prepared with minor modification as follows (Samsu *et al*. 2014): NaNO_3_ (5.5 g/L), MgSO_4_ 7H_2_0 (0.5 g/L), KCl (1.0 g/L), K_2_HPO_4_ (0.3 g/L), trace elements (1.0 mL) that composed of Na_3_C_6_H_5_O_7_ (2.0 g/L), FeCl (0.28 g/L), ZnSO_4_ (1.40 g/L), CoCl_3_ (1.20 g/L), CuSO_4_ (1.20 g/L), MnSO_4_ (0.80 g/L). The concentration of all agro-industrial waste substrates was fixed to 3% (v/v). MSM was sterilised by autoclaving at 121°C for 15 min.

### Production of Biomass and Rhamnolipid in Shake Flask

A vial of *P. aeruginosa* USM-AR2 seed culture was propagated in nutrient broth (NB) for 24 h before being inoculated at 2% (v/v) aseptically into a 250 mL Erlenmeyer flask containing 50 mL MSM, each supplemented with respective agro-industrial waste substrate tested. The culture was then incubated at 27 ± 2°C and agitated at 200 rpm. The experiment was conducted in triplicates for each substrate. Samples were taken every 12 h interval for bacterial growth and rhamnolipid production determination.

### Growth Quantification

Cells were harvested via centrifugation at 10,000 ×*g* for 5 min (Eppendorf Centrifuge 5424, Eppendorf AG, Hamburg, Germany). The supernatant was collected for rhamnolipid quantification. Cells were resuspended in acetone to remove the adhering oil residue (for oil substrates only), vortexed, centrifuged again and washed with distilled water to remove residual medium. The cell pellet was resuspended in 3 mL distilled water and bacterial growth was determined at OD_540_ nm with a spectrophotometer (Genesys 20, Model 4001-04, Thermo Scientific, USA). The biomass, expressed in cell dry weight (g/L), was determined by comparison to a pre-constructed calibration curve.

### Rhamnolipid Quantification: Orcinol Assay

Rhamnolipid quantification was carried out based on Nur Asshifa *et al*. (2017) with minor modification. Rhamnose was used as an indirect reference to quantify rhamnolipid. Extracellular glycolipid concentration was evaluated by measuring the concentration of rhamnose: To 0.3 mL sample, 2.7 mL of a solution containing 0.19% orcinol (in 53% H_2_SO_4_) was added. After incubation for 50 min at 70°C, the samples were cooled at room temperature and the optical density (OD_421_ nm) was measured using a spectrophotometer. The rhamnolipid concentration was calculated from a pre-constructed calibration curve prepared with L-rhamnose by comparing the readings with those of rhamnose standards between 0.5 g/L and 1.5 g/L expressed as rhamnose equivalents (RE) (g/L).

### Gas Chromatography Mass Spectrophotometry Analysis of Oil Waste Substrates

Oil palm based agro-industrial wastes in this study were analysed by gas chromatography mass spectrometry (GC-MS) for fatty acid composition identification. The peaks that represent fatty acid in the chromatogram were determined by referring to the fatty acid methyl esters (FAME) Mass Spectral Library. The GC-MS analysis was carried out using GCMS-QP2010 Ultra (Shimadzu, Japan) with a PBX 70 (0.25 mm × 60 m × 0.25 μm) column by passing helium as the carrier gas at a flow rate of 3.0 mL/min. The column temperature was maintained at 100°C for 2 min and thereafter increased gradually at a rate of 2°C/min to 230°C (Ichihara & Fukubayashi 2010).

### Reducing Sugar Determination by 3,5-dinitrosalicylic acid (DNS) Assay

DNS assay (Miller 1959) was used to measure glucose (Glucose RTU kit, Biomérieux, France) in sugar-based and starch-based agro-industrial substrates. Samples were mixed with 2 mL DNS solution and boiled for 10 min. The change in colour was measured using a spectrophotometer at a wavelength of 540 nm. The assay was done in triplicates. The reading was then compared to a calibration curve constructed using glucose at known concentrations.

## RESULTS

### Sugar and Fatty Acid Content of Waste Substrates

**Figure 1:**
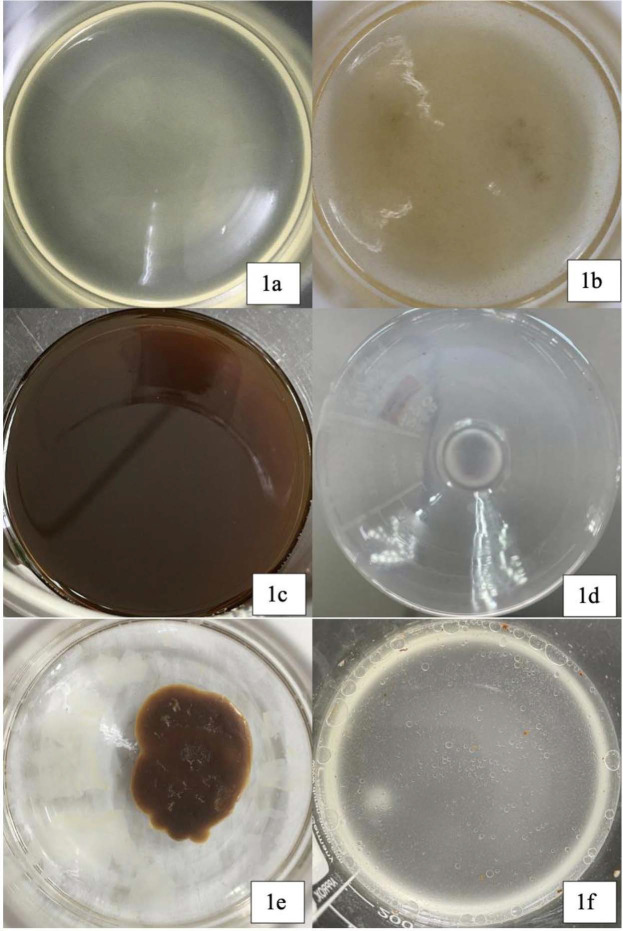
Agro-industrial waste appearance at 3% (v/v) in MSM before fermentation at room temperature. (a) POME; (b) EFB steam effluent; (c) sugarcane molasses; (d) rice washing water; (e) PSO; and (f) OMC.

Table 1 shows the glucose content in different sugar substrates after mixed in MSM. The physical appearance of each substrate are shown in Fig. 1 (1a to 1f). Based on Table 1, the fermentation using OMC shows the highest glucose concentration at 1.19 g/L, followed by those using sugarcane molasses and rice water with the lowest concentration of glucose.

**Figure 2:**
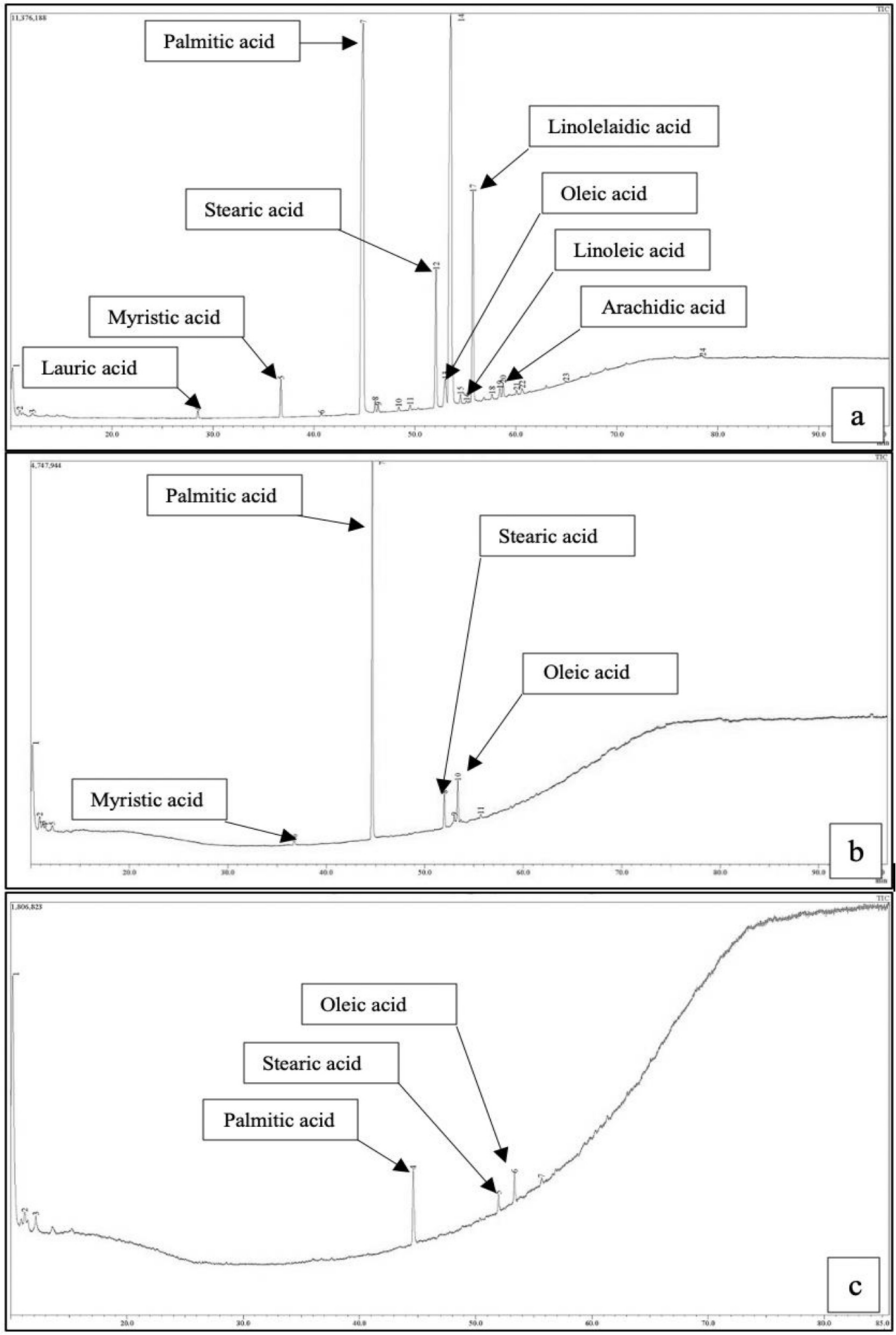
Chromatograms of different oil palm-based waste substrates by GC-MS analysis; (a) PSO; (b) EFB steam effluent; and (c) POME.

For oil palm based substrate, the fatty acid content was analysed using GC-MS FAME analysis. Figs. 2a to 2c show the chromatograms for each oil palm substrate. A broad peak was also observed at the end of the retention time for each substrate. The diversity of fatty acids within the substrate is tabulated in Table 2, showing PSO having the highest diversity of fatty acid species followed by EFB steam effluent and POME.

### Growth and Rhamnolipid Production in Shake Flask

The growth and rhamnolipid production of *P. aeruginosa* USM-AR2 on all six agro industrial wastes are shown in Table 3. The highest growth and rhamnolipid production were observed with PSO at 15.33 g/L and 0.97 g/L, respectively. EFB steam effluent resulted in a biomass concentration of 1.07 g/L with lower rhamnolipid at 0.58 g/L than that of POME. In this study, all three oil palm substrates exhibited higher cell growth and rhamnolipid production than sugar substrate that resulted in 0.16 g/L of biomass using rice water and 0.13 g/L of rhamnolipid by OMC.

### Growth and Rhamnolipid Production at Different Substrates Concentration

Table 4 shows the growth and rhamnolipid production by *P. aeruginosa* USM-AR2 grown on three oil palm substrates at 5% (v/v) substrate concentration. An increased in cell growth and rhamnolipid production were observed on all three substrates with PSO showing the highest biomass of 20.78 g/L and rhamnolipid concentration of 1.07 g/L at 5% (v/v) substrate concentration. Moreover, an increase on productivity was observed where PSO showed the highest productivity of 0.007 g/L/hr followed by POME at 0.005 g/L/hr, while the lowest was EFB steam effluent at 0.0003 g/L/hr. EFB steam effluent is a poor substrate for rhamnolipid production giving the lowest rhamnolipid concentration of 0.25 g/L although the substrate concentration was increased to 5% (v/v).

## DISCUSSION

### Cell Growth and Rhamnolipid Production in Shake Flask

Various agro-industrial substrates collected were evaluated as potential carbon sources for rhamnolipid production by *P. aeruginosa* USM-AR2. The ability of *P. aeruginosa* cells to grow on various substrates including sugar and oil substrates has been reported in previous studies (Santosa *et al*. 2020; Li *et al*. 2011). In this study, *P. aeruginosa* USM-AR2 was observed to grow on all six substrates tested as the main carbon source with different rhamnolipid production as presented in Table 3. The six substrates were classified into two groups which were oil palm based agro-industrial substrates and, saccharide substrates. The composition of oil palm based agro-industrial waste substrates was analysed via GC-MS analysis whereas, for saccharides agro-industrial waste, DNS assay were carried out to analyse the glucose content in the substrates.

Each agro-industrial waste posed different challenges during handling, preparation or fermentation process due to different properties and complex liquid-solid behaviour. As each substrate was generated from its own unique process, a particular preservation and preparation steps need to be taken into consideration (Nasaruddin *et al*. 2016). PSO was autoclaved separately from the growth medium to prevent the substrate from clumping and subsequently creating a semi-solid mixture that reduced contact surface area (Thinagaran & Sudesh 2019). On the other hand, POME and EFB steam effluent must be sieved first to separate the liquid portion from the suspended solid palm fibres that came from leaves, trunk, decanter cake, empty fruit bunches, seed shells and fibre from the mesocarp, introduced during the extraction (Bukhari *et al*. 2020). Figs. 1a to 1f show each agro-industrial substrate in aqueous culture medium in a shake flask before fermentation. Unlike homogenous substrates within the medium, shown in Figs. 1a to 1d, PSO formed a solid clump on the surface of the liquid medium as shown in Fig. 1e. In contrast, droplets of oil were observed on the surface of medium for OMC, as shown in Fig. 1f. Waste substrates were prone to spoilage and contamination during improper storage. Hence, all substrates collected were stored at 4°C except for OMC and rice washing water, which were used fresh upon collection from the market (Rahman *et al*. 2018).

*Pseudomonas aeruginosa* USM-AR2 showed a lower rhamnolipid production using saccharide substrates, whereas PSO, an oil substrate, supported the highest cell growth at 15.33 g/L and rhamnolipid production at 0.97 g/L (Table 3). PSO is a dark brown solid at room temperature. However, in this experiment, as the fermentation progressed, it gradually dispersed in the fermentation broth, turning the medium into milky yellow with a thin layer of oil sludge before being emulsified. Previous studies conducted by Jamal *et al*. (2011) and Wan Nawawi *et al*. (2010) that used this substrate had shown the potential of PSO for biosurfactant production and demonstrated the biosurfactant activity via surface tension reduction of the liquid growth medium to 36.2 mN/m and 23.44 mN/m, respectively.

GC-MS analysis of the fatty acid content showed all three oil palm based agro-industrial wastes, namely EFB steam effluent, POME, and PSO, have palmitic acid as the highest concentration compared to other fatty acids, as reported by other studies (Matinja *et al*. 2019; Ahmad *et al*. 2016). The peaks presented in Figs. 2a to 2c were determined upon comparing with the FAME Mass Spectral Library. Based on Figs. 2a to 2c, towards the end of the retention time, a broad peak was observed in all three substrates with the largest one observed in POME. This was suspected to be the unresolved complex compound within the substrates. The peak in POME was larger due to its high impurity content given that it is the final waste to be discharged into the waste pond. Similar unresolved compound was observed by Ganapathy *et al*. (2019) when treating POME using *Meyerozyma guilliermondii*.

Based on Table 2, PSO showed the highest composition of fatty acids, consisting of palmitic acid (C_16_) (54.48%), stearic acid (C_18_) (11.73%), and linoleic acid (C_18_) (9.02%). In contrast, EFB steam effluent contained only four types of fatty acids with palmitic acid (C_16_) as the most predominant fatty acid (62.75%). In addition, three types of fatty acids were detected in POME with palmitic acid being the highest at 15.85%. This finding is in accordance with those in the previous studies by Radzuan *et al*. (2017) and Wafti *et al*. (2012) that reported high free fatty acid (FFA) content of PSO. The free fatty acid such as palmitic acid, oleic acid and linoleic acid are generally utilised for cell growth (Lan *et al*. 2015). They are known to be one of the important precursors in the fatty acid de novo synthesis which contributes to produce the hydrophobic moiety of rhamnolipid (Gutiérrez-Gómez *et al*. 2019). Hence, the higher composition of fatty acid in PSO could lead to the high cell biomass and rhamnolipid production compared to that of the other two substrates.

For saccharide waste substrates, a DNS assay was carried out to measure the glucose content of the substrates. Glucose content was analysed as glucose serves as one of the main precursors of rhamnolipid production other than fatty acid in its biosynthetic pathway. Based on Table 1, the highest glucose content was obtained in OMC at 1.19 g/L, which also showed a higher rhamnolipid production compared to that of the other sugar substrates (sugarcane molasses and rice water). Low glucose level in sugarcane molasses is in accordance with previous reports where sugarcane molasses typically contains 17%–25% water, 30%–40% sucrose, 5%–12% fructose and 4%–9% glucose (Clarke 2003; Jamir *et al*. 2021). Moreover, sugarcane molasses was diluted before being added into MSM to reduce its viscosity and allow filter sterilisation using 0.22 μm filter membrane, which simultaneously resulted in low glucose concentration. In addition, a study by Borah *et al*. (2015) has shown rhamnolipid produced by *P. aeruginosa* SS14 was higher when using glucose compared to that when using sucrose.

Rhamnolipid production is higher for substrate with higher glucose because of the rhamnolipid synthesis that depends on two pathways. The first is the fatty acid de novo synthesis. The other pathway is the activated (dTDP-) rhamnose pathway. In this case, OMC provided the glucose needed for the production of the intermediate precursor for rhamnose synthesis (Tiso *et al*. 2016). This finding contradicted the study that used sugarcane molasses as the main carbon source which reported rhamnolipid production as high as 4.47 g/L, at higher temperature and larger inoculum size (38°C and 7% inoculum size) (Santos *et al*. 2010). It is important to highlight that the maximum rhamnolipid production by using OMC is still lower compared to the that by using the immiscible fatty acid rich substrate, PSO. This further supports that *P. aeruginosa* culture favours water-immiscible substrates over miscible sugar substrates for growth and metabolite production (Tan & Li 2018).

Among all the tested substrates, rice washing water exhibited the lowest production of rhamnolipid which was 0.03 g/L despite showing higher biomass production than sugarcane molasses and OMC. Starch contains amylose or amylopectin molecules that require a specific enzyme system to assist the transport of the compounds into the cell. Therefore, the bacterial culture needs to secrete enzymes such as amylase and oligo-1,6-glucosidase in order to break the starch into smaller glucose subunits before they can enter the cell through glycolytic pathway (Petrova *et al*. 2013).

### Growth and Rhamnolipid Production at Different Substrates Concentration

Subsequently, substrates that gave the highest rhamnolipid production among all the agro-industrial substrates tested were subjected to further experiment of different substrate concentration. All three of the oil palm wastes, namely EFB steam effluent, PSO and POME gave higher rhamnolipid production compared to that of sugar and starch-base substrates. An increase of PSO concentration to 5% (v/v) resulted in the highest biomass and rhamnolipid production which was 20.78 g/L and 1.07 g/L, respectively, due to the higher carbon and nutrient content to support higher cells growth and product formation. This is also supported by its productivity of 0.007 g/L/h indicating PSO utilisation was more efficient for rhamnolipid production (Table 4). In addition, a finding by Sathi *et al*. (2016) on the effect of substrate concentrations of mango kernel oil when used as sole or co-substrate for rhamnolipid production by *P. aeruginosa*, reported an increase in rhamnolipid production at 1.81 g/L when the substrate concentration was increased from 0.5% to 1%. Further increase in the substrate concentration to 1.5% resulted in a decrease of rhamnolipid production to 1.12 g/L.

## CONCLUSION

The present work demonstrates the potential of several Malaysian agro-industrial waste substrates for rhamnolipid production in *P. aeruginosa* USM-AR2 fermentation. Oil palm-based agro-industrial waste substrates resulted in a higher rhamnolipid production compared to saccharide substrates. Among this, PSO gave the highest production of rhamnolipid compared to EFB steam effluent and POME. Further study is aimed to apply the potential substrate for rhamnolipid production in a larger scale reactor system. The use of selected agro-industrial waste can serve as a cheap alternative substrate while minimising waste disposal problem for environmental management.

## Figures and Tables

**Table 1:** Glucose content in different sugar substrates.

Agro-waste	Glucose concentration (g/L)
Rice water	< 0.001
Sugarcane molasses	0.240
Overly matured coconut (OMC)	1.190

**Table 2:** Fatty acid composition in oil palm-based substrates.

Substrate	Fatty acid	Percentage
Palm sludge oil (PSO)	Lauric acid	0.75
	Myristic acid	3.32
	Palmitic acid	54.48
	Stearic acid	11.73
	Oleic acid	0.90
	Linoleic acid	9.02
	Linolelaidic acid	8.45
	Arachidic acid	0.92
Empty fruit bunch (EFB) steam effluent	Myristic acid	1.38
	Palmitic acid	62.75
	Stearic acid	4.85
	Oleic acid	1.01
Palm oil mill effluent (POME)	Palmitic acid	15.85
	Stearic acid	3.13
	Oleic acid	7.64

**Table 3:** Biomass and rhamnolipid production in shake flask fermentation with different agro-industrial waste substrates.

Agro-industrial substrates	Biomass (g/L)	Rhamnolipid (g/L)
Sugarcane molasses	0.12 ± 0.009	0.10 ± 0.013
Overly-matured coconut (OMC) water	0.13 ± 0.016	0.13 ± 0.006
Rice washing water	0.16 ± 0.007	0.03 ± 0.002
Empty fruit bunch (EFB) steam effluent	1.07 ± 0.158	0.23 ± 0.014
Palm oil mill effluent (POME)	0.25 ± 0.101	0.58 ± 0.071
Palm sludge oil (PSO)	15.33 ± 0.364	0.97 ± 0.072

**Table 4:** Effect of different substrate concentrations on biomass and rhamnolipid production in shake flask fermentation.

Agro-industrial substrate	3% (v/v)			5% (v/v)		

	Biomass (g/L)	Rhamnolipid (g/L)	Productivity (g/L/hr)	Biomass (g/L)	Rhamnolipid (g/L)	Productivity (g/L/hr)
Empty fruit bunch (EFB) steam effluent	1.07 ± 0.158	0.23 ± 0.014	0.0008	1.44 ± 0.055	0.25 ± 0.063	0.0003
Palm oil mill effluent (POME)	0.25 ± 0.101	0.58 ± 0.071	0.003	0.45 ± 0.026	0.62 ± 0.080	0.005
Palm sludge oil (PSO)	15.33 ± 0.364	0.97 ± 0.072	0.006	20.78 ± 1.045	1.07 ± 0.041	0.007
